# Peer Pressure = Explosive Consequences: A Case Report of Toxic Ingestion of Cyclonite (C-4) Explosive on a Dare

**DOI:** 10.5811/cpcem.2020.10.49241

**Published:** 2021-01-12

**Authors:** Joshua D. Whitesides, Major Nathaniel Turner, Susan Watts, Sarah A. Watkins

**Affiliations:** * Texas Tech University Health Sciences Center El Paso, Department of Emergency Medicine, El Paso, Texas; † William Beaumont Army Medical Center, Department of Emergency Medicine, El Paso, Texas; ‡ West Texas Poison Control Center, El Paso, Texas

**Keywords:** Cyclonite, C4, RDX, plastic explosive, seizures, military explosives

## Abstract

**Introduction:**

We present a case of intentional ingestion of a piece of plastic explosive in a military patient that resulted in tonic-clonic seizure and gastrointestinal illness.

**Case Report:**

Although uncommon, such ingestions have been reported in military journals since the Vietnam War. Access to plastic explosives is generally limited to military personnel, and non-military medical providers may not be familiar with treatment of acute intoxication.

**Conclusion:**

It is imperative to refresh awareness and provide education to heighten suspicion and broaden differential diagnosis for patients presenting with new onset syncope or seizure, particularly in the military population.

## INTRODUCTION

Cyclotrimethylenetrinitramine (common names cyclonite, Research Development Explosive [RDX]) is the predominant explosive ingredient in Composition-4 (C-4) plastic explosive used extensively since World War II by the military in the United States (US) and other countries.^1^,^2^ It is a white solid with a putty-like consistency (Image). Previous studies have revealed that soldiers may intentionally ingest C-4 for two reasons: to elicit intoxication with symptoms similar to ethanol ingestion, or to induce illness for secondary gain.^1^,^3^ There are also reports of unintentional occupational exposure in the manufacture of C-4.

Reported symptoms of ingestion include nausea, vomiting, diarrhea, seizures, postictal coma, lethargy, dysrhythmia, and myoclonus. Cardiac abnormalities such as tachycardia and quadrigeminal rhythm have also been reported; however, the exact mechanism has not been elucidated.^4^ In addition to ingestion, exposure due to inhalation was reported, on average, four times per month between December 1968–December 1969 when C-4 explosive was used to heat food rations, since it gives off intense heat without exploding.^5^ It is important to note that both unintentional and intentional exposures to C-4 have a common presentation: new onset seizure in a non-epileptic patient. 6

## CASE REPORT

A 20-year-old male, active duty US Army Combat Engineer was brought to the emergency department (ED) via ambulance after ingesting a small piece of C-4, with a volume estimated to be about 1.2 cubic inches, or three-quarter ounce. As a combat engineer, his duties involved detonation of explosives, including C-4, in support of combat operations. During a lull between training iterations at a demolition range, he was dared by a colleague to eat a 4 × 1 centimeter piece of plastic explosive. Immediately following the ingestion, he began to have nausea and a headache. Approximately 30 minutes later, he sustained a witnessed, generalized tonic-clonic seizure lasting two minutes. His unit members contacted their medical support and he was transported to the nearest military ED.

Per emergency medical services, he was alert and oriented to person, place, time, and situation with reassuring vital signs. No antiepileptic medications were administered prior to arrival at the ED. In the ED, the patient reported feeling confused, dizzy, and weak for about 60 minutes following the seizure. He complained of new onset of lower back pain. His initial vital signs were as follows: blood pressure 149/71 millimeters of mercury; heart rate 96 beats per minute; respiration 15 breaths per minute; and his rectal temperature was normal. His physical examination was remarkable for warm skin, hyperreflexia, and non-sustained bilateral lower extremity clonus.

Electrocardiogram (ECG) showed sinus tachycardia without evidence of ischemia, and borderline corrected QT interval prolongation was reported; however, no specific measurement was provided in the patient’s record. Radiographs of the chest, thoracic spine, and lumbar spine showed no acute abnormalities, and there was no mention of radiopaque foreign bodies. Computed tomography of the head showed no evidence of acute hemorrhage, edema, or mass effect.

When it became clear the patient had been exposed to C-4, the West Texas Regional Poison Center was consulted. Additional laboratory tests were recommended to assess for signs of organ damage and metabolic aberrations: urinalysis for hematuria; blood gas for methemoglobinemia; comprehensive metabolic panel for elevated anion gap metabolic acidosis, acute kidney or liver injury and hypokalemia; and creatine kinase to assess for rhabdomyolysis. Supportive care was recommended for seizures and nausea. Twenty-four hour observation was recommended as well.

The patient received lorazepam 1 milligram (mg) intravenously (IV), ketorolac 30 mg IV, and 1 liter (L) normal saline bolus. Laboratory results were significant for leukocytosis of 16.4 10^3^/microliters (uL) (reference range 4.5 to 11.0 x10^9^/uL); elevated D-dimer of 2003 nanograms per milliliter (ng/mL) D-dimer units (reference range <250 ng/mL); and elevated lactate at 2.41 millimoles (mmol)/L (reference range 0.5–1 mmol/L).

**CPC-EM Capsule**

What do we already know about this clinical entity?

Ingestion of the plastic explosive Composition-4 (C-4), whether intentional or unintentional, may result in neurologic, gastrointestinal, renal, and cardiac effects.

What makes this presentation of disease reportable?

Ingestion of C-4 is an uncommon practice. Yet it appears cyclically in military medical literature, and non-military medical providers should be made aware.

What is the major learning point?

Ingestion of C-4 commonly results in seizures, nausea, and vomiting. Seizures associated with C-4 toxicity respond to standard doses of benzodiazepines.

How might this improve emergency medicine practice?

Our goal was to raise awareness that certain patient populations such as young males in the military might ingest plastic explosive and suffer C-4 toxicity.

He was admitted to the intensive care unit (ICU) for monitoring. On admission, the consulting neurologist recommended doses of valproate 20 mg per kilogram (kg) and levetiracetam 40 mg/kg IV for seizure prophylaxis. In addition, the inpatient team elected to order two 16-ounce doses of polyethylene glycol oral solution and magnesium citrate for gastrointestinal decontamination. There was no report of C-4 found in rectal effluent. He remained on seizure precautions and had resolution of gastrointestinal symptoms, and no further seizure activity or other neurologic symptoms.

Once medically cleared, he was seen by a behavioral health specialist per standard Army protocol. He met face-to-face with a licensed clinical social worker (LCSW) and was evaluated using a battery of seven standardized psychological screening tools routinely used in the US Army Behavioral Healthcare Clinic. There were no concerning results and he was cleared by the LCSW and returned to full duty after his 24-hour observation period.

At follow-up with his primary care provider four days later, he denied any recurrence of symptoms and did not have any further complaints or seizure-like activity.

## DISCUSSION

On our review of the literature, we found that fewer than 10 case reports of human C-4 ingestion have been published in the last 20 years and all published cases involve military personnel.^4^,^8^,^9^ The cases describe symptoms ranging from acute gastrointestinal illness to severe neurological sequelae. As seen with our patient, the most commonly reported symptom from exposure was new onset of seizures; these have been reported even from occupational exposures where appropriate handling and packaging of materials have been performed.^1^ Toxic effects have also been reported in animals. For example, in 2008 a two-year-old male Labrador retriever working in explosive detection ingested C-4 resulting in tonic-clonic seizures.^7^

The practice of purposeful ingestion of C-4 occurs disproportionately in young males. Analysis of case reports from 1969–2019, including both occupational and intentional exposures, found 31 were male, and none were female. Mean age was 26 years old. The average time from ingestion to onset of symptoms was approximately seven hours, with a range of 30 minutes to 16 hours.^4^,^8^–^9^ Ingestion quantities were inconsistently reported; however, a minimum ingestion of 1.58 grams to a maximum of 180 grams were reported. Of note, seizures were reported in 100% of cases.^1^–^6^,^8^–^9^

Pathophysiologically, seizures after C-4 ingestion are a direct result of central nervous system toxicity. Animal models have shown that cyclonite, the toxic ingredient in C-4, binds *in vivo* to the gamma-aminobutyric acid A (GABA_A_) chloride channel at the picrotoxin binding site with the same or greater affinity than pentylenetetrazol, acting as a non-competitive inhibitor of chloride conductance and resulting in seizure activity.^8^,^10^–^12^ The treatment of status epilepticus from C-4 ingestion is medication with competitive GABA_A_ receptor binding, such as benzodiazepines, barbiturates, and propofol. The management of a patient suspected to have ingested C-4 is primarily symptomatic and supportive. An algorithmic approach including first establishing a patent airway followed by prompt ECG to analyze for arrhythmias is imperative. Administration of activated charcoal is then suggested in patients not at risk for aspiration, followed by standard doses of benzodiazepines to prevent and treat seizures.^13^ Fluid resuscitation and monitoring of urine output are indicated because acute renal injury is common, and a soldier presenting from a training event is at high risk of volume depletion. After stabilizing the patient, ICU admission for close observation is reasonable.

## CONCLUSION

Exposure to C-4, whether occupational or intentional, is most notable for new onset seizures but can also result in neurologic, renal, and gastrointestinal abnormalities. After standard initial management including airway control to prevent aspiration, the next steps include medications to control seizures, and then assessment for end organ damage. Young adult males who may be susceptible to peer pressure and ingest C-4 as a rite of passage are a particularly at-risk population. It is imperative that emergency providers consider C-4 ingestion as a possible cause for unexplained first-time seizures in populations with access to the compound.

## Figures and Tables

**Image:**
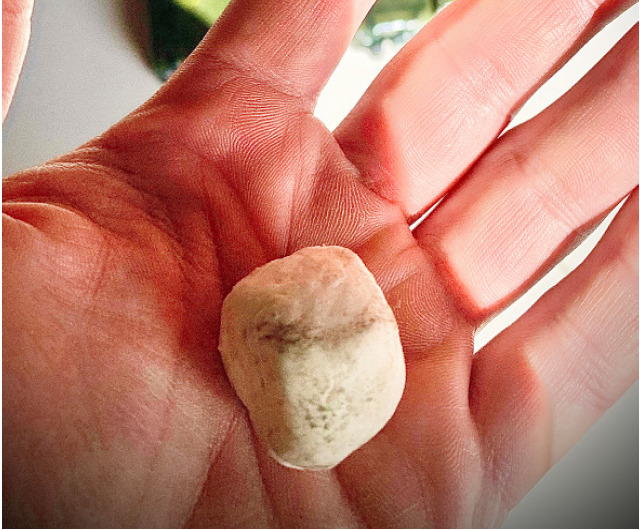
The plastic explosive compound C-4 is a white solid with a putty-like consistency.
